# Antibacterial coating of tooth surface with ion-releasing pre-reacted glass-ionomer (S-PRG) nanofillers

**DOI:** 10.1016/j.heliyon.2021.e06147

**Published:** 2021-02-02

**Authors:** Kayoko Mayumi, Hirofumi Miyaji, Saori Miyata, Erika Nishida, Tomokazu Furihata, Yukimi Kanemoto, Tsutomu Sugaya, Kanako Shitomi, Tsukasa Akasaka

**Affiliations:** a Department of Periodontology and Endodontology, Faculty of Dental Medicine, Hokkaido University, N13 W7, Kita-ku, Sapporo, Hokkaido, 060-8586, Japan; b Division of Periodontology and Endodontology, School of Dentistry, Health Sciences University of Hokkaido, 1757 Kanazawa, Tobetsu-cho, Ishikari-gun, Hokkaido, 061-0293, Japan; c Department of Biomedical Materials and Engineering, Faculty of Dental Medicine, Hokkaido University, N13 W7, Kita-ku, Sapporo, Hokkaido, 060-8586, Japan

**Keywords:** *Actinomyces naeslundii*, Animal model, Antibacterial activity, Anti-inflammatory, Class II furcation defect, Ion-releasing, Periodontitis, Root dentin, *Streptococcus mutans*, Surface pre-reacted glass-ionomer (S-PRG) filler

## Abstract

**Objectives:**

Surface pre-reacted glass-ionomer (S-PRG) fillers release antibacterial borate and fluoride ions. We fabricated nanoscale S-PRG fillers (S-PRG nanofillers) for antibacterial coating of tooth surfaces and assessed the antibacterial effects of this coating in vitro. In addition, we creating a canine model of periodontitis to evaluate the effectiveness of S-PRG nanofiller application on tooth roots and improvement of periodontal parameters.

**Methods:**

Human dentin blocks were coated with S-PRG nanofiller (average particle size: 0.48 μm) and then characterized by scanning electron microscopy (SEM), energy dispersive X-ray spectrometer (EDX), and ion-releasing test. Antibacterial effects of dentin blocks coated with S-PRG nanofiller were examined using bacterial strains, *Streptococcus mutans* and *Actinomyces naeslundii.* Next, we created an experimental model of periodontitis in furcation of premolars of beagle dogs. Then, S-PRG nanofiller coating was applied onto exposed tooth root surfaces. Periodontal parameters, gingival index (GI), bleeding on probing (BOP), probing pocket depth (PPD), and clinical attachment level (CAL), were measured from baseline until 4 weeks. In addition, bone healing was radiographically and histologically examined.

**Results:**

SEM and EDX revealed that S-PRG nanofillers uniformly covered the dentin surface after coating. Dentin blocks coated with S-PRG nanofiller showed ion-releasing property, bacterial growth inhibition, and sterilization effects. In the experimental periodontitis model, S-PRG nanofiller coating significantly reduced clinical inflammatory parameters, such as GI (P < 0.01) and BOP (P < 0.05), compared to uncoated samples. In addition, PPD and CAL significantly decreased by S-PRG nanofiller coating (2 weeks: P < 0.05; 3 and 4 weeks: P < 0.01), suggesting the improvement of periodontitis. Micro-CT and histology revealed that bone healing of furcation defects was enhanced by S-PRG nanofiller coating.

**Conclusion:**

S-PRG nanofiller coating provides antibacterial effects to tooth surfaces and improves clinical parameters of periodontitis.

## 1 Introduction

The progression of inflammatory periodontal disease causes tooth loss and diminishes human health to decrease mastication function. Daily tooth brushing only cannot completely remove bacterial biofilms in deep periodontal pockets [1, 2]. In addition, antibiotics are slightly effective against infections associated with bacterial biofilms attached to the tooth surface [3]. Hence, the development of a novel antimicrobial strategy is needed. Recently, as a local drug delivery system, antibacterial nanomaterials to modify the tooth surface have been experimentally tested. Metallic nanoparticles showed significant antibacterial effects against oral bacterial cells through antibacterial metallic ion release [4, 5, 6]. Therapy comprising nanoparticle coating of tooth surfaces is likely beneficial for prevention and improvement of periodontal disease. However, various properties of metal nanomaterials, such as loading dose, particle size, and cytotoxicity, must be further examined to exert stable, long-term, and biosafe activities.

Surface pre-reacted glass-ionomer (S-PRG) filler has been reported as an ion-releasing, biofunctional glass filler to enhance the physical properties and bioactivities of dental materials, such as composite resin and endodontic sealer [7, 8, 9]. The surface of the core glass of the S-PRG filler possesses a thin glass-ionomer phase, therefore, it can rapidly release ions related to glass-ionomer phase: borate, fluoride, sodium, strontium, aluminum, and silicate [10, 11]. A previous in vitro report revealed that dental material including S-PRG fillers showed long-term ion-releasing profiles for at least 90 days [12]. In particular, two ions released from S-PRG filler, borate and fluoride, exhibit antibacterial effects. Moreover, borate exhibits bacteriostatic effects, that is, suppression of further bacterial biofilm formation of various bacterial strains [13, 14]. Anti-inflammatory ophthalmic agent of boric acid is clinically applied to irrigate human eyes [15]. Fluoride ion also shows antibacterial activity, as well as acid-resistant and tooth remineralization effects [16]. Eluting solution of S-PRG fillers including fluoride and borate ions significantly inhibits proliferation and subsequent sugar metabolism of *Streptococcus mutans,* a major pathogen of human dental caries, and protects hydroxyapatite from acidizing to repair tooth [17, 18]. Furthermore, S-PRG filler suppresses growth of *Candida albicans*, which is related to oral candidiasis [19]. Hence, we hypothesized that S-PRG filler coating of exposed tooth surfaces induced by periodontitis would provide long-term antibacterial activity via ion release to subsequently improve periodontal inflammatory responses.

In general, nano-sized particles tend to be aggregated by attractive force because of the high surface area to volume ratio [20]. Hence, we attempted to prepare nanoscale S-PRG fillers (S-PRG nanofillers) to coat tooth surfaces. In addition, we assessed the antibacterial effect of human tooth surfaces coated with S-PRG nanofillers against two oral bacteria, *S. mutans* and *Actinomyces naeslundii*. These bacteria are associated with early biofilm formation [21, 22, 23]. In addition, preclinical examination of tooth coating therapy with S-PRG nanofillers was carried out in an experimental canine model of periodontitis by premolar furcation. Infected tooth roots were cleaned and coated with S-PRG nanofillers to assess the improvement of clinical periodontitis parameters. In addition, bone healing of furcation bone defects was radiographically and histologically examined.

## 2 Materials and methods

### 2.1 Fabrication of S-PRG nanofiller

S-PRG filler was fabricated according to previous reports [7, 24]. Frit of fluoroboroaluminosilicate glass (composition: 21.6 wt% SiO_2_, 21.6 wt% Al_2_O_3_, 16.6 wt% B_2_O_3_, 27.2 wt% SrO, 2.6 wt% Na_2_O, and 10.4 wt% F) was produced by melting at 1400 °C for 2 h. After dry- and wet-grounding of glass frit, treatment of polysiloxane (SiO_2_ content: 16 wt%; degree of condensation: 2–6) and polyacrylic acid (polymer content: 13.0 wt%, average molecular weight: 52,000) was subsequently subjected to finally obtain S-PRG filler.

S-PRG nanofiller was prepared by sedimentation with S-PRG filler. S-PRG filler was mixed with distilled water (DW) at 1:1 ratio (w/w) and stirred well. After standing for 16 h, the supernatant was collected to obtain the nanoscale range glass particles; namely, S-PRG nanofiller. S-PRG fillers and nanofillers were characterized by scanning electron microscopy (SEM; S-4000, Hitachi Ltd., Tokyo, Japan) with accelerating voltage of 10 kV after coating with a thin layer of Pt–Pd. In addition, the particle size of S-PRG nanofiller was measured by using a particle size distribution (PSD) measuring instrument (Microtrac MT 3300 EX II, MicrotracBEL Inc., Osaka, Japan).

### 2.2 Preparation of dentin blocks

Dentin blocks were prepared from extracted vital third molars of patients over 20 years old treated at the dental department of Hokkaido University Hospital. The use of human teeth in this study was conducted in compliance with the Declaration of Helsinki and approved by the Institutional Review Board of Hokkaido University Hospital for Clinical Research (approval No. 17-222). Informed consent was obtained from all participants at Hokkaido University Hospital. The tooth root was shaped using a diamond disk (Horico diamond disk 87xFSI, Horico, Berlin, Germany) and sandpaper (#600 and #2000) to fabricate the dentin block (5 × 5 × 1 mm). After ultrasonic cleaning with DW for 5 min to remove the surface smear layer, the dentin block was obtained for evaluation.

### 2.3 Coating of dentin blocks with S-PRG nanofiller and characterization

S-PRG fillers and nanofillers were dispersed in and adjusted by water to obtain 1 wt% dispersion, respectively. The dentin block was then immersed into the S-PRG nanofiller dispersion for 3 min to coat the block surface with nanofillers and then washed 3 times with DW. After drying, the dentin block surface was observed by field emission SEM (JSM-6500F, JEOL Ltd., Tokyo, Japan) equipped with an energy dispersive X-ray spectrometer (EDX) and compared to uncoated dentin block surface.

For the ion-releasing test, S-PRG nanofiller coated dentin blocks were immersed in DW (5 mL) for >24 h. Fluoride-, borate-, and strontium-containing DW were measured using a fluoride electrode (model 9609BN; Orion pH/ion meter, model 720A, Thermo Fisher Scientific, Waltham, MA, USA) after determining calibration curves (standard solutions: 0.02, 0.1, 1, 20 ppm) and an inductively coupled plasma atomic emission spectrometer (ICPS-8000, Shimadzu Corporation, Kyoto, Japan), respectively.

We carried out ultrasonic cleaning of S-PRG filler or nanofiller coated dentin and observed the morphology. Dentin blocks were coated with S-PRG fillers or nanofillers and then washed 3 times with DW. After that, dentin blocks were ultrasonically washed using an ultrasonic cleaner (45 kHz, VS-100 III, AS ONE Corporation., Ltd, Osaka, Japan) for 1 or 10 s. After drying and Pt–Pd coating, dentin block surfaces were characterized by SEM and compared with residual fillers on the dentin block surface.

### 2.4 Antibacterial properties of S-PRG nanofiller coated dentin

We assessed the antibacterial activity of S-PRG nanofiller coated dentin. S-PRG nanofiller coated and uncoated dentin blocks were prepared as described above. Each dentin block was placed into a well of a 48-well plate. The suspension of facultative anaerobic bacteria, *S. mutans* ATCC 35668 or *A. naeslundii* ATCC 27039, was seeded onto the dentin block (final concentration: 1 × 10^7^ CFU/mL) and cultured in brain heart infusion (BHI) broth (Pearlcore®, Eiken Chemical, Co., Ltd., Tokyo, Japan) supplemented with 0.1% antibiotic (gramicidin D and bacitracin, FUJIFILM Wako Pure Chemical Corporation, Osaka, Japan) and 1% sucrose for *S. mutans* or actinomyces broth (BBL™ Actinomyces Broth, Becton, Dickinson and Company, Franklin Lakes, NJ, USA) for *A. naeslundii*. After 24-h culture periods, dentin blocks were removed and washed with fresh BHI broth using a vibrator to collect bacterial cells attached to the dentin block. Broth containing bacterial cells were diluted 10-fold in fresh broth, spread onto BHI agar plates (Eiken Chemical Co., Ltd), and incubated at 37 °C for 24 h. The number of collected bacteria was subsequently calculated by colony count.

In addition, each dentin block incubated with *S. mutans* was observed by SEM following conventional procedure and stained by the LIVE/DEAD BacLight Bacterial Viability Kit (Thermo Fisher Scientific). Live bacteria were stained with SYTO9 to produce green fluorescence and dead bacteria with compromised membranes were stained with propidium iodide to produce red fluorescence. Observation was carried out using a fluorescence microscope (BioRevo BZ-9000, Keyence Corp., Osaka, Japan). The intensity of green and red fluorescence was measured using ImageJ 1.41 (National Institutes of Health, Bethesda, MD, USA).

### 2.5 Creation of experimental periodontal defect in dog

To investigate the clinical effects of S-PRG nanofiller coating, we conducted a preclinical experiment using beagle dogs. Four female beagle dogs, aged 12–16 months and weighing approximately 10 kg, were used for this experiment. Animal experiments were performed in accordance with the institutional animal use and care regulations of Hokkaido University (Animal Research Committee of Hokkaido University) and approved by the Animal Research Committee of Hokkaido University (approval number 17-93). Surgical procedures were performed under general anesthesia with medetomidine hydrochloride (0.04 mg/kg, Domitor®; Nippon Zenyaku Kogyo Co., Ltd., Fukushima, Japan), butorphanol tartrate (0.15 mg/kg, Vetorphale®; Meiji Seika Pharma Co., Ltd., Tokyo, Japan) and midazolam (0.15 mg/kg, Dormicum® Injection 10 mg; Astellas Pharma Inc., Tokyo, Japan) under local anesthesia with 2% lidocaine hydrochloride with 1:80,000 epinephrine (Xylocaine Cartridge for Dental Use, Dentsply Sirona K.K., Tokyo, Japan).

First, periodontitis was experimentally induced in beagle dogs as previously described [25]. A buccal mucogingival flap of mandibular premolars was reflected to expose alveolar bone. Class II buccal furcation defects (5 mm in height, 3 mm horizontally) were created at the second, third, and fourth premolars. After planing of the exposed root, the furcation defect was filled with silicone impression material (Examixfine regular type, GC Corporation, Tokyo, Japan) to induce infection to the root surface and inflammatory responses by dental plaque (Figure S1A). The flap was then repositioned and sutured. At 4 weeks after surgery, silicone impression material was removed and exposure of the root surface of premolars was confirmed.

### 2.6 S-PRG nanofiller coating of tooth roots and periodontal examination

One week after removing silicone impression material, periodontal examination at baseline was carried out by one blinded examiner. Clinical parameters gingival index (GI), bleeding on probing (BOP), probing pocket depth (PPD), and clinical attachment level (CAL) at two buccal areas close to the furcation defect (distal area in the mesial root and mesial area in the distal root of the premolars) were measured (Table 1) as described previously [26]. BOP was calculated as the percentage of bleeding pockets from total measured pockets. Subsequently, all periodontal pockets of the mandibular premolars were ultrasonically cleaned to remove dental plaque and calculus from the root surface using an ultrasonic scaler (Enac-Osada ultrasonic unit, Osada Electric Co., Ltd., Tokyo, Japan). Premolars were then randomly divided into two areas: left or right premolar rows (including second, third, and fourth premolars). In the experimental area, S-PRG nanofiller (1 wt%) dispersion was injected into the periodontal pocket using a syringe until it overflowed to coat the root surface with S-PRG nanofillers (Figure S1B). Dispersion of S-PRG nanofiller was added with 20 wt% glycerin to improve injection handling. In the control area (uncoated), no dispersion was applied. Periodontal parameters (GI, BOP, PPD, and CAL) were continuously recorded by one blinded examiner every week for 4 weeks after baseline. No dogs received mechanical or chemical plaque control through the experimental period.

**Table 1:** Clinical parameters of periodontal disease.

GI	**0**	Normal
	**1**	Mild inflammation, slight color change and edema, no bleeding.
	**2**	Moderate inflammation, redness, edema, bleeding on probing.
	**3**	Severe inflammation, marked redness and edema, ulceration and spontaneous bleeding.
BOP	Presence or absence of bleeding within 10 s following probing of the pocket.	
PPD	The distance between the gingival margin and the bottom of the probeable pocket.	
CAL	The distance between the cementoenamel junction and the bottom of the probeable pocket.	

GI, gingival index; BOP, bleeding on probing; PPD, probing pocket depth; CAL, clinical attachment level.

### 2.7 X-ray assessment and histological observation

After clinical observation and subsequent euthanization using an overdose of sodium pentobarbital under general anesthesia, tissue blocks including the furcation defect of mandibular fourth premolars were removed, fixed in 10% buffered formalin, and radiographically assessed for bone healing by X-ray microcomputed tomography (micro-CT) scanner (Latheta LCT-200, Hitachi, Ltd., Japan). From the captured micro-CT images, the radiopacity of the furcation area of premolars was measured using ImageJ and the percent bone volume (BV/TV) was calculated.

Subsequently, for histological examination, tissue blocks were decalcified in 10% formic acid and embedded in paraffin wax following conventional procedures. Sections along the mesio-distal plane were prepared, stained with Masson's trichrome, and observed using light microscopy.

### 2.8 Statistical analysis

Statistical analysis was performed by the Mann–Whitney *U* Test. *P* values < 0.05 were considered statistically significant. All statistical procedures were performed using SPSS 11.0 (IBM Corporation, Armonk, NY, USA).

## 3 Results and discussion

### 3.1 Characterization of S-PRG nanofillers

**Figure 1:**
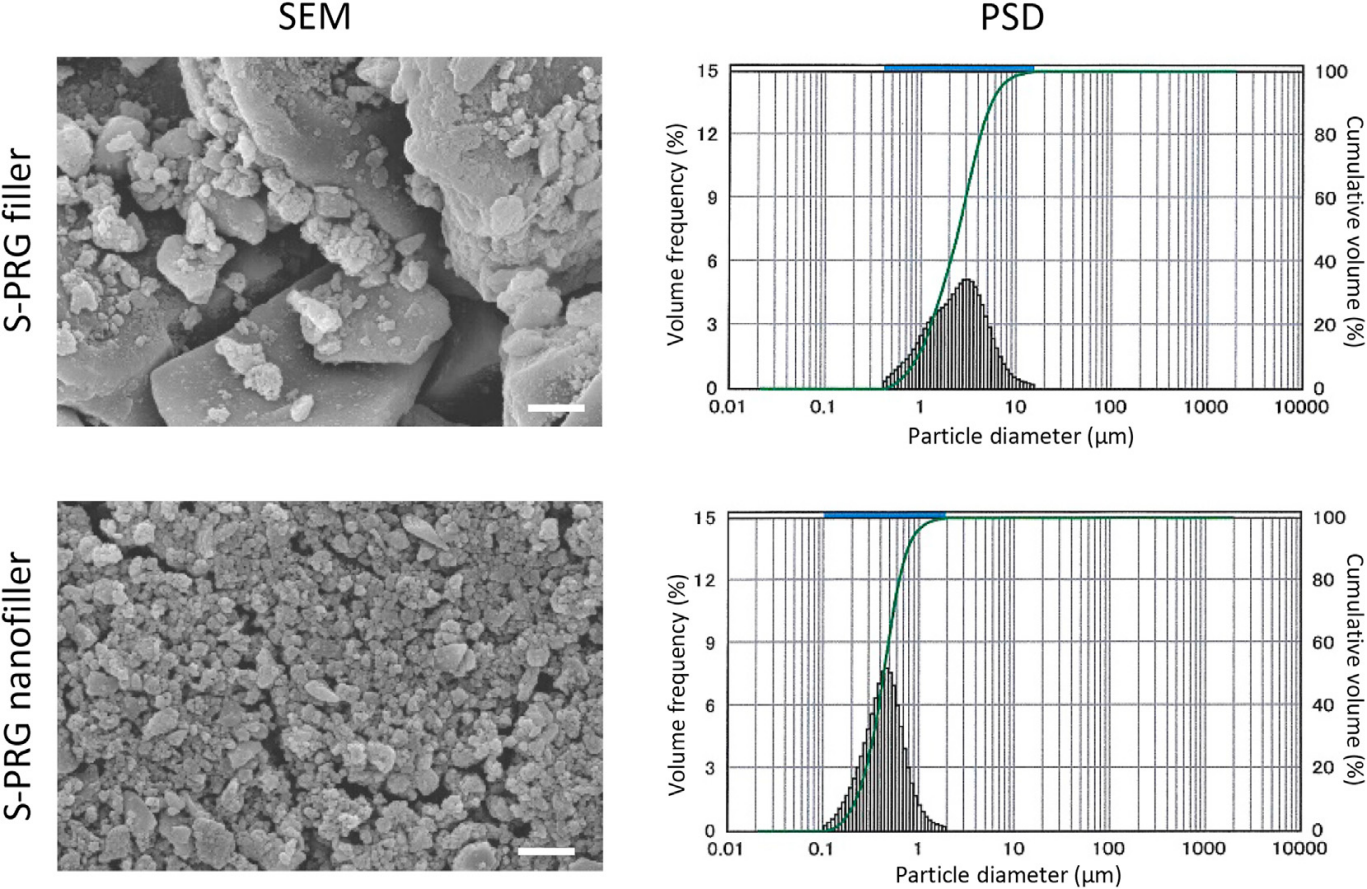
SEM micrographs and PSD measurements of S-PRG fillers and nanofillers. Scale bar = 1 μm. SEM, scanning electron microscopy; S-PRG, surface pre-reacted glass ionomer; PSD, particle size distribution.

Using the sedimentation procedure, the fraction of S-PRG nanofiller was obtained from S-PRG filler dispersion. SEM images and PSD measurements of S-PRG fillers and nanofillers are shown in Figure 1. S-PRG fillers contained nano- and microparticles. In contrast, well-regulated nanoparticles were found in S-PRG nanofillers. The size distribution peak of S-PRG fillers and nanofillers was at 3 and 0.5 μm particle diameter, respectively. In addition, the mean volume diameter of S-PRG fillers and nanofillers was 2.95 and 0.48 μm, respectively.

### 3.2 S-PRG nanofiller coating of dentin surfaces

**Figure 2:**
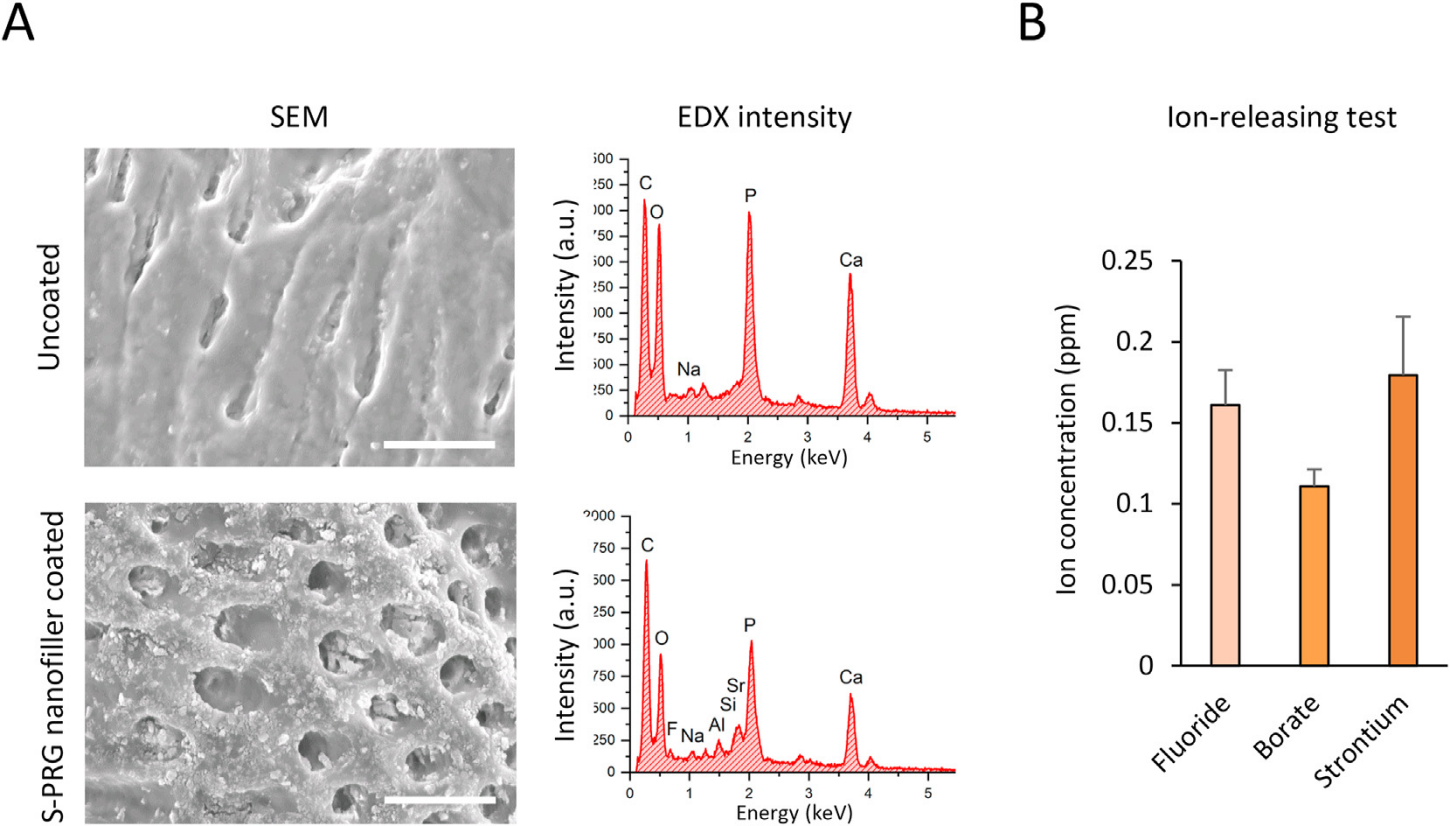
Characterization of S-PRG nanofiller coated dentin. (A) SEM micrographs and EDX intensity of S-PRG nanofiller coated or uncoated dentin block surfaces. Scale bar = 10 μm. (B) Amount of released ions from S-PRG nanofiller coated dentin (n = 3, mean ± SD). EDX, energy dispersive X-ray spectrometry; SD, standard deviation; SEM, scanning electron microscopy; S-PRG, surface pre-reacted glass-ionomer.

Figure 2A shows surfaces of dentin blocks coated with S-PRG nanofiller or uncoated using SEM and EDX analyses. S-PRG nanofiller particles uniformly covered the dentin surface compared to uncoated dentin. Furthermore, nanofillers frequently filled open dentinal tubules. In EDX analysis, elemental peaks for F, Al, Si, and Sr (Si and Sr were overlapped), which are associated with S-PRG filler components, were particularly identified on the dentin blocks coated with S-PRG nanofillers compared with uncoated dentin blocks. Elements C, P, and Ca of dentin substrate were detected in all samples. The ion releasing test showed that fluoride, borate, and strontium ions were detected from dentin coated with S-PRG nanofillers (Figure 2B). Fluoride, borate, and strontium ion concentrations were 0.16, 0.11, and 0.18 ppm, respectively.

**Figure 3:**
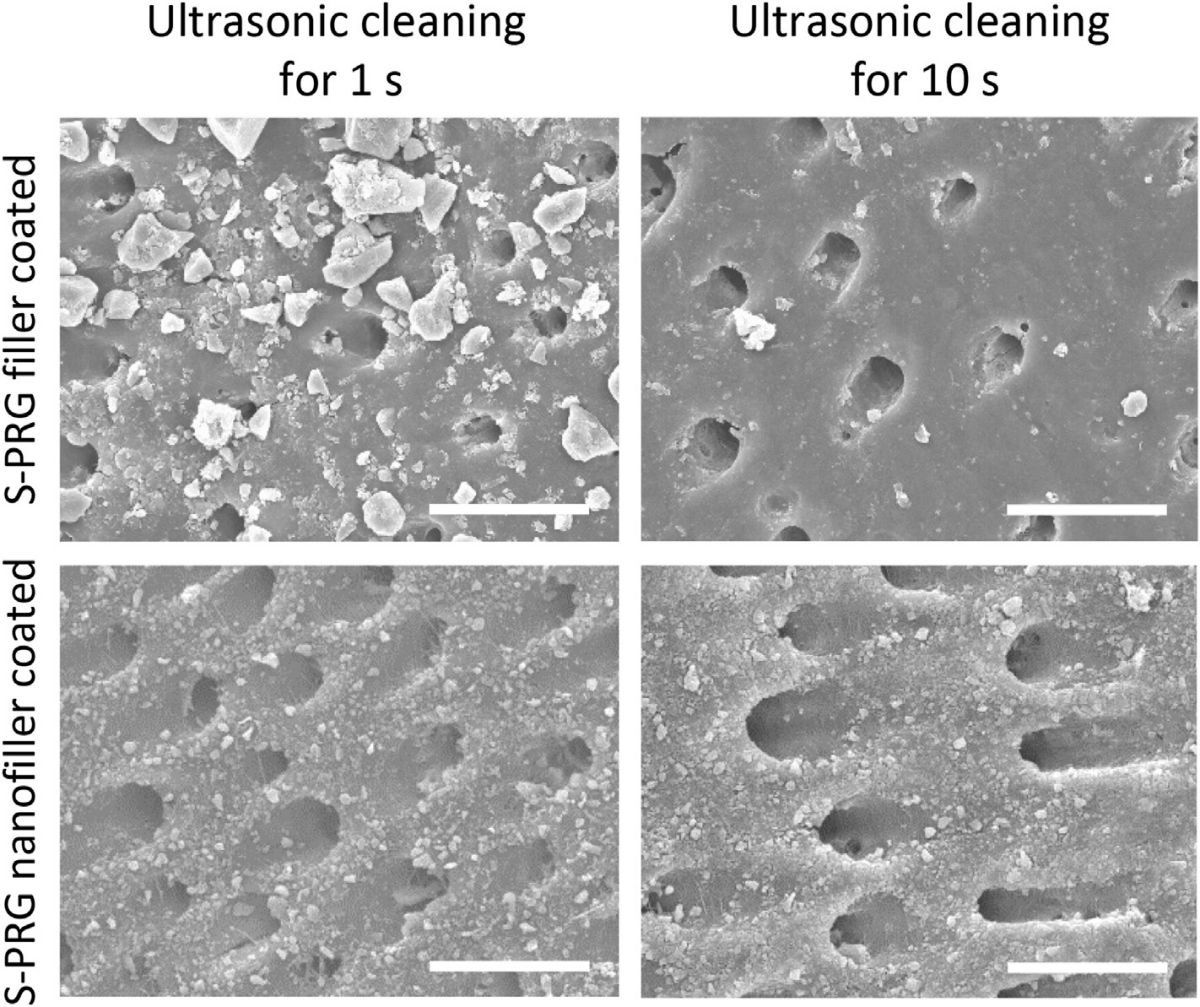
Ultrasonic cleaning test. SEM micrographs of S-PRG filler or nanofiller coated dentin surface after ultrasonic cleaning for 1 and 10 s. Scale bar = 10 μm. SEM, scanning electron microscopy; S-PRG, surface pre-reacted glass-ionomer.

The results of ultrasonic cleaning of S-PRG filler or nanofiller coated dentin are shown in Figure 3. Microscale particles of S-PRG filler were observed on the dentin block surface after ultrasonic cleaning for 1 s, however, they disappeared after ultrasonic cleaning for 10 s. In contrast, S-PRG nanofillers remained on the dentin block surface after ultrasonic cleaning for 10 s, similar to results of ultrasonic cleaning for 1 s. From this result, S-PRG nanofiller may easily adhere to dentin. Nanoscale particles tend to aggregate by various attractive forces [27]. Some reports explained that nanoscale material for drug delivery systems exhibits bioadhesive capability via electrostatic potential [28, 29]. Previous reports explored tooth constructs, such as enamel, dentin, and cementum, and demonstrated these structures possess electrostatic potential [30, 31]. The mechanism of S-PRG nanofiller attachment to dentin likely involves attractive forces such as van der Waals and/or electrostatic interactions. The bioadhesive property of S-PRG nanofiller could be advantageous for clinical operation in periodontal treatment.

### 3.3 Antibacterial assessments of S-PRG nanofiller coated dentin

**Figure 4:**
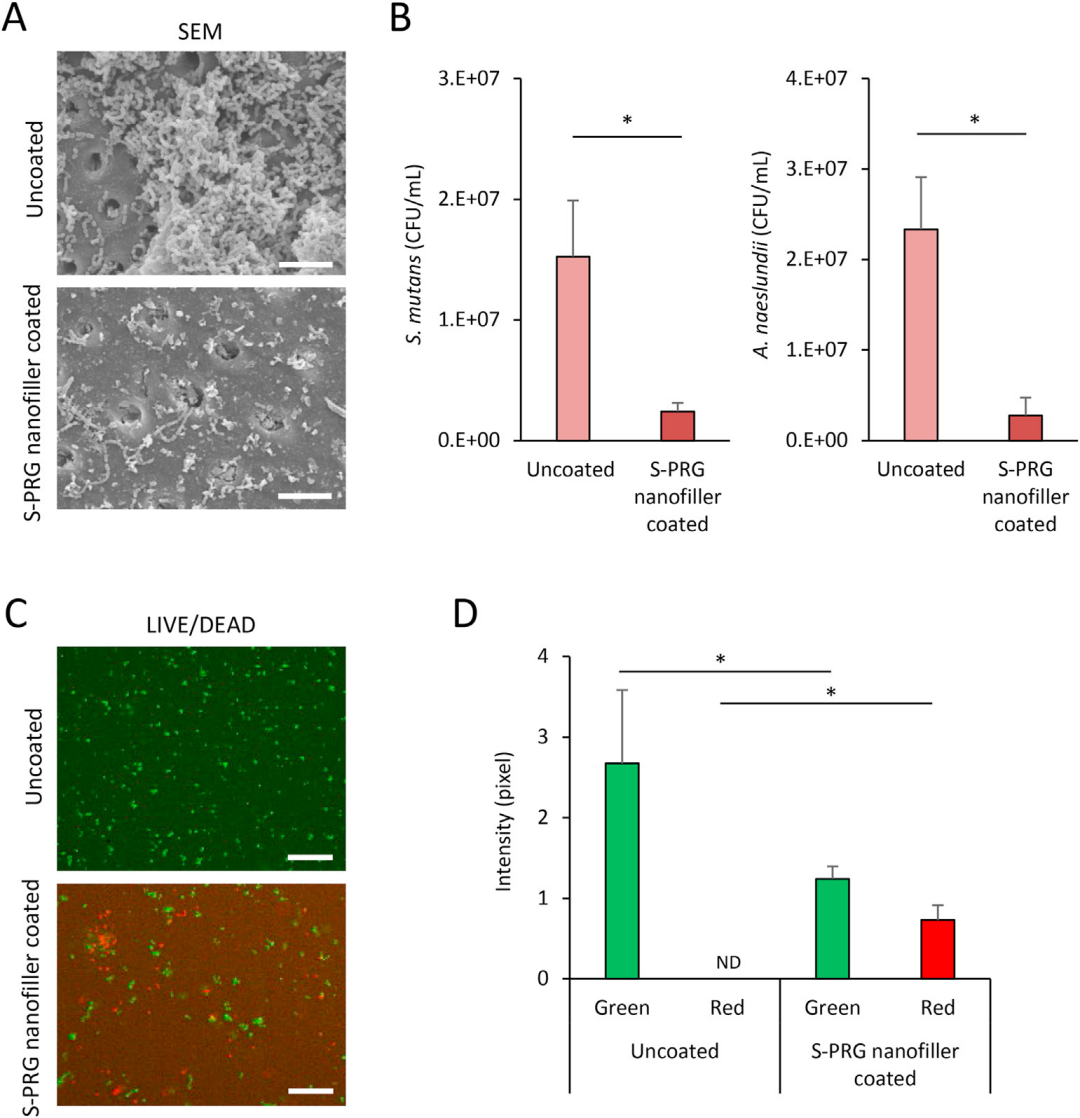
Antibacterial assessments of S-PRG nanofiller coated dentin. (A) SEM micrographs of S-PRG nanofiller coated or uncoated dentin surfaces incubated with *S. mutans* for 24 h. Scale bar = 10 μm. (B) Bacterial counts of *S. mutans* and *A. naeslundii* (n = 4, mean ± SD); ∗*P* < 0.05. (C) LIVE/DEAD BacLight staining of *S. mutans* after 24-h incubation. Scale bar = 20 μm. (D) Intensity of live (green) and dead (red) bacteria (n = 3, mean ± SD). ∗*P* < 0.05. *A. naeslundii*, *Actinomyces naeslundii*; CFU, colony-forming units; ND; not detected; SD, standard deviation; SEM, scanning electron microscopy; *S. mutans*, *Streptococcus mutans;* S-PRG, surface pre-reacted glass-ionomer.

SEM images of dentin blocks seeded with *S. mutans* for 24-h incubation are shown in Figure 4A. Uncoated dentin had marked *S. mutans* colonization. In contrast, dentin blocks coated with S-PRG nanofiller showed slight bacterial accumulation and biofilm formation. *S. mutans* and *A. naeslundii* counts were significantly reduced to approximately 1/10th by S-PRG nanofiller coated dentin compared with uncoated dentin (P < 0.05) (Figure 4B). LIVE/DEAD BacLight staining also demonstrated mainly dead *S. mutans* cells (shown by red fluorescence) on dentin blocks coated with S-PRG nanofillers. In contrast, uncoated dentin blocks rarely showed dead *S. mutans* cells (Figure 4C). The intensity of green and red fluorescence was 2.67 and 0 in the uncoated group, and 1.24 and 0.73 in the S-PRG nanofiller coated group, respectively. A significant decrease in live cells and increase in dead cells were observed in the S-PRG nanofiller coated group compared to the uncoated group (P < 0.05) (Figure 4D).

S-PRG nanofiller coated dentin block possess fluoride and borate ion release capability (Figure 2B). Fluoride and borate ions inhibit the metabolism of bacterial cells as antibacterial substances [32, 33]. Dentin blocks coated with S-PRG nanofiller showed inhibition activity against both *S. mutans* and *A. naeslundii* in the present study, suggesting that antibacterial effects were provided by fluoride and borate ions released from S-PRG nanofillers attached to dentin blocks. Furthermore, nanoparticles penetrate the cell membrane via interaction forces [34], thus, nanofillers might be imported into the bacterial cell body to directly destruct internal bacterial organs, in addition to ion releasing effects. S-PRG filler may also exhibit anti-caries properties. The tooth-restorative effects of acid resistance and remineralization are mediated by fluoride ions of S-PRG filler [35, 36]. The tooth surface from which bacterial biofilms have been removed by S-PRG nanofillers may be reinforced by fluoride ions released from them to inhibit tooth demineralization. Further research is needed to elucidate the precise mechanisms underlying multiple functions of S-PRG nanofiller coating in addition to antibacterial activity, especially caries prevention.

### 3.4 Periodontal healing after S-PRG nanofiller coating

**Figure 5:**
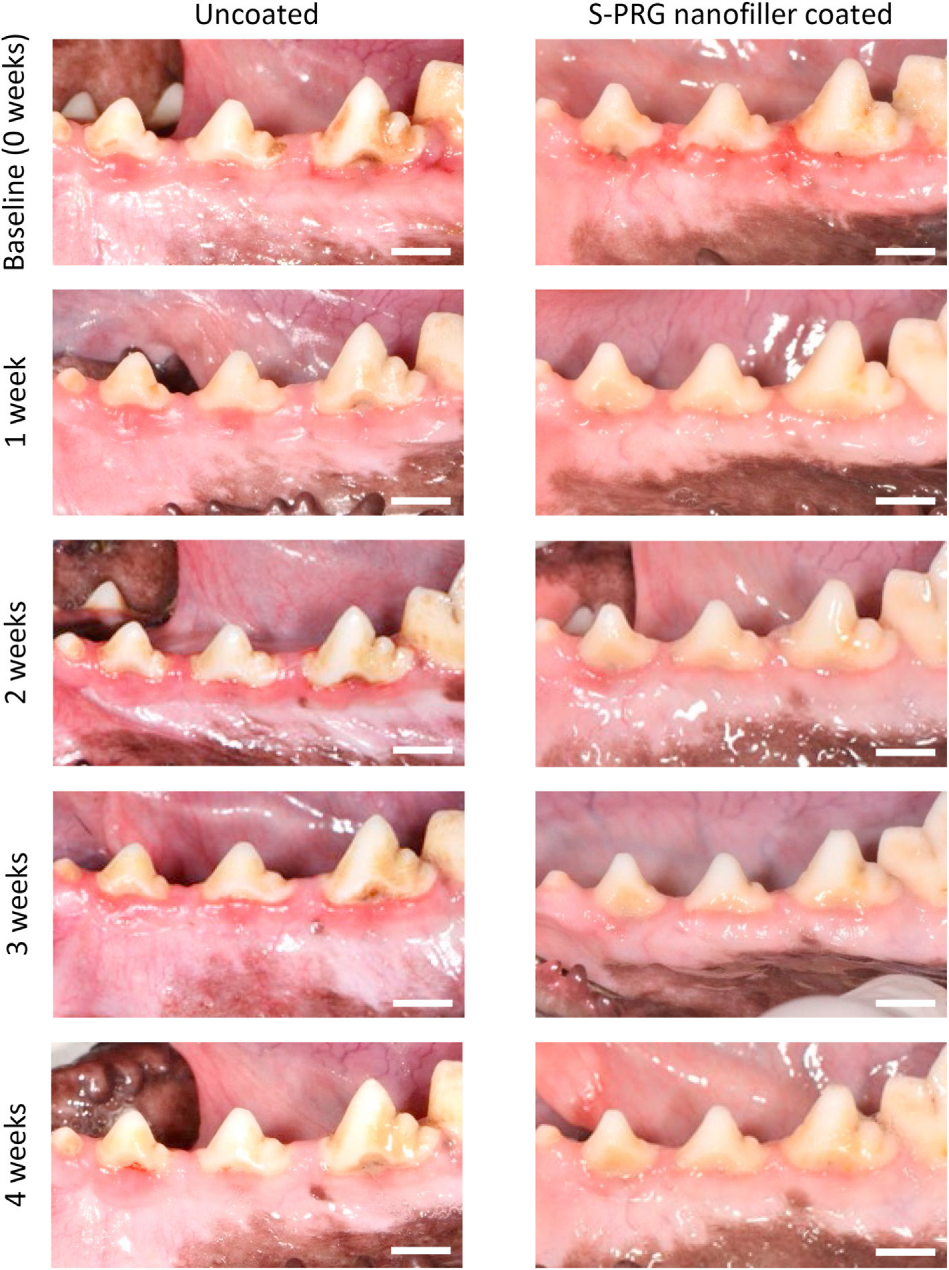
Digital photographs of gingival tissue around second, third, and fourth premolars from baseline to 4 weeks in uncoated and S-PRG nanofiller coated groups. Scale bar = 1 cm. S-PRG, surface pre-reacted glass-ionomer.

Figure 5 shows digital photographs of gingiva around premolars. At baseline, both S-PRG nanofiller coated and uncoated groups showed redness of marginal gingiva, suggesting that gingivitis was elicited by the detention of impression material and invasion of dental plaque. After 1 week, gingival tissue redness remained in the uncoated group, however, S-PRG nanofiller coating eliminated inflammation until 4 weeks after coating. In the uncoated group, gingival tissue redness was frequently found up to 4 weeks after baseline.

**Figure 6:**
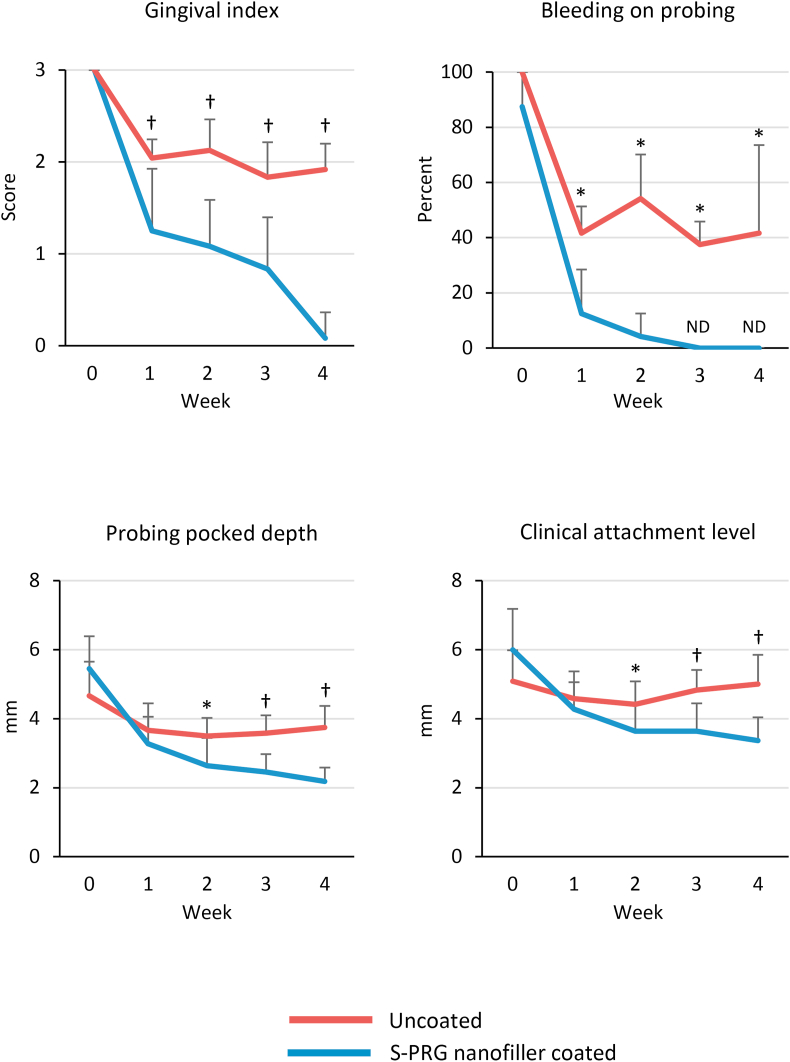
Periodontal parameters from baseline to 4 weeks of S-PRG nanofiller coated or uncoated groups (n = 12, mean ± SD). ∗*P* < 0.05, †*P* < 0.01, vs. S-PRG nanofiller coated group. ND; not detected; S-PRG, surface pre-reacted glass-ionomer.

Results of periodontal examination are shown in Figure 6. S-PRG nanofiller coating improved the parameters of inflammatory response. The mean GI score at baseline was 3 in both S-PRG nanofiller coated and uncoated groups. The mean GI score in the uncoated group was approximately 2 from 1 to 4 weeks, whereas the S-PRG nanofiller coated group had a GI score of 1 at 1 week, which decreased to approximately 0 at 4 weeks. There were significant differences in GI scores between S-PRG nanofiller coated and uncoated groups at 1, 2, 3, and 4 weeks (P < 0.01 for all). The incidence of BOP at baseline was approximately 90–100% in both groups. After 1 week, the BOP decreased in the uncoated group, which remained at approximately 40–50% until 4 weeks. In contrast, the incidence of BOP in the S-PRG nanofiller coated group dramatically decreased to 10% after 1 week and no BOP was observed after 3 weeks. Significant differences were found at 1, 2, 3, and 4 weeks between S-PRG nanofiller coated and uncoated groups (P < 0.05). PPD and CAL showed similar trends during the observation period. PPD and CAL values in the S-PRG nanofiller coated group decreased in a time-dependent manner compared to the uncoated group. At 4 weeks, PPD and CAL were 3.75 and 5 mm in the uncoated group and 2.18 and 3.36 mm in the S-PRG nanofiller coated group, respectively. Significant differences were found after 2 weeks between S-PRG nanofiller coated and uncoated groups (2 weeks: P < 0.05; 3 and 4 weeks: P < 0.01).

These results suggested that S-PRG nanofiller coat of the root surface improved periodontal inflammatory parameters in dog. In vitro antibacterial testing showed that S-PRG nanofiller coated dentin inhibits *S. mutans* and *A. naeslundii* activity. In addition, S-PRG nanofillers could adhere to the human root cementum as well as dentin (Figure S2). Hence, tooth roots coated with S-PRG nanofillers likely inhibit biofilm regrowth in periodontal pockets, subsequently reducing periodontal inflammation. In the uncoated group, biofilm reconstruction may have occurred, although periodontal pockets were cleaned by the ultrasonic scaler. In general, periodontal inflammatory responses are strongly related to bacterial action in the periodontal pocket [37, 38]. Previous study revealed that *S. mutans* stimulated coaggregation of obligate anaerobic periodontal bacteria, members of red and orange complex, such as *P. gingivalis* and *F. nucleatum* [21, 39]. In addition, *A. naeslundii* is a well-known primary colonizer of the tooth surface to subsequently form the dental biofilm [40]. Detailed changes in bacterial flora in the periodontal pocket following S-PRG nanofiller application as periodontal treatment should be elucidated in future studies.

### 3.5 Bone healing assessments of furcation defect

**Figure 7:**
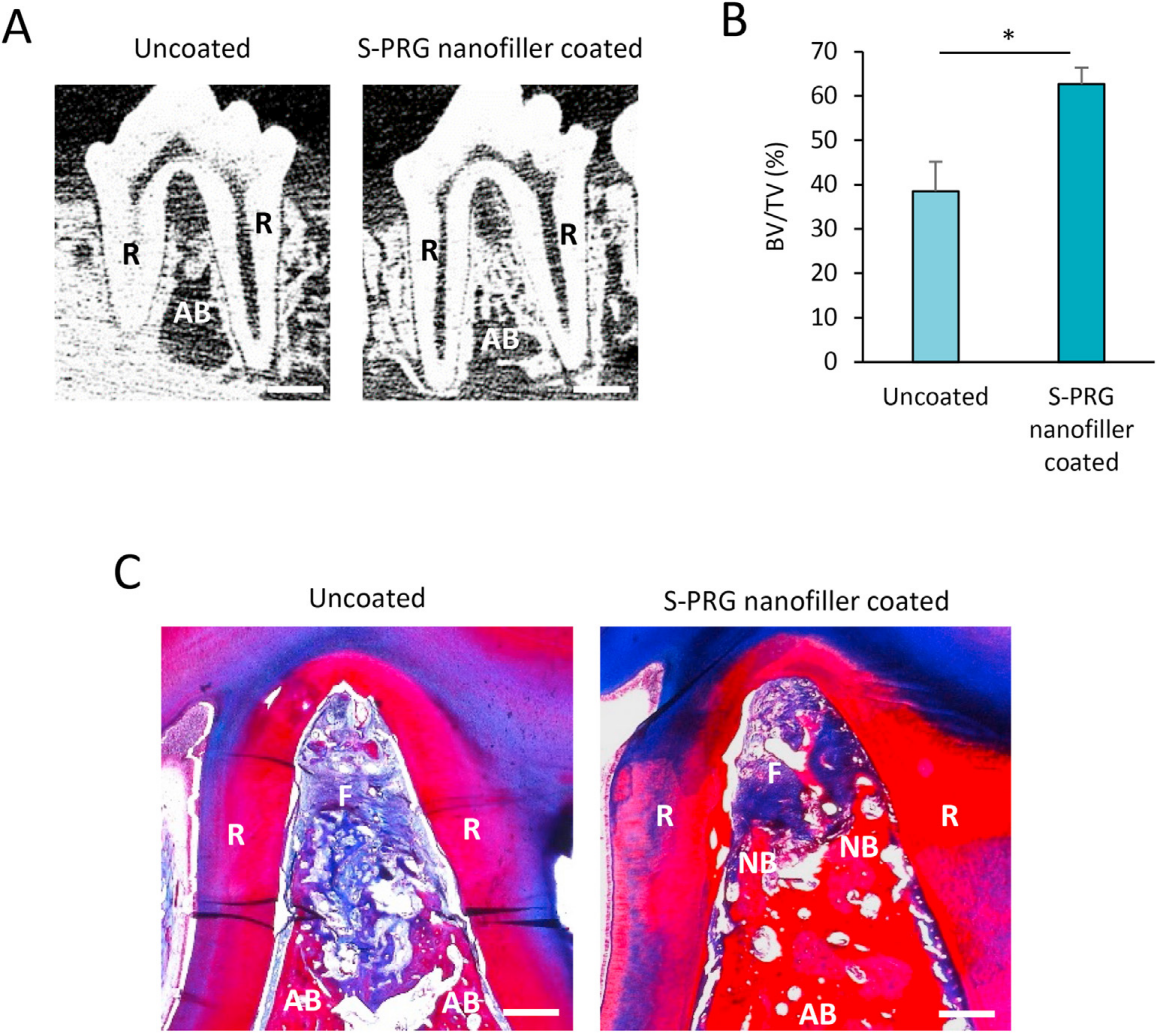
Micro-CT and histological analysis. (A) Micro-CT images of premolar at 4 weeks. Scale bar = 5 mm. (B) Percent bone volume (BV/TV) of furcation defect at 4 weeks (n = 4, mean ± SD). ∗*P* < 0.05. (C) Histological findings of furcation defect of premolar at 4 weeks. Scale bar = 1 mm. AB, alveolar bone; F, fibrous tissue; NB, newly formed alveolar bone; R, tooth root; S-PRG, surface pre-reacted glass-ionomer.

Micro-CT images of premolars including the furcation defect at 4 weeks are shown in Figure 7A. Furcation defects receiving S-PRG nanofiller coating demonstrated increased radiopacity along the tooth root compared to uncoated defects. BV/TV of S-PRG nanofiller coated group was significantly higher than that of the uncoated group (62.7% and 38.5%, respectively, P < 0.05) (Figure 7B). Histological specimens are shown in Figure 7C. In the uncoated group, bone and periodontal tissue reconstruction were slight. The furcation region was occupied by inflammatory connective tissue. In contrast, the S-PRG nanofiller coated group frequently showed bone formation in the furcation region.

From these results, it seemed likely that periodontal tissue healing proceeded due to the diminishment of inflammation by S-PRG nanofiller application. Iwamatsu-Kobayashi et al. demonstrated that eluting solution of S-PRG fillers, including multiple ions, prevented tissue destruction in a murine model of ligature-induced periodontal disease [41]. The authors also detected that diseased periodontal tissue could incorporate fluoride, borate, and strontium ions transferred from S-PRG filler eluting solution. In addition to the antibacterial activity of fluoride and borate ions, strontium ions from S-PRG filler may exhibit an anti-inflammatory effect in the immunosuppression mechanisms in vivo. Strontium ion reduces interleukin-6 expression, tumor necrosis factor-α, and generation of reactive oxygen species [42, 43, 44]. In addition, previous reports showed that strontium ions are strongly related to bone inductive activity. Application of strontium ions stimulates osteogenic differentiation of human adipose-derived stem cells [45, 46] and mesenchymal stem cells and bone formation in vivo [47, 48]. Furthermore, fluoride ions also enhance osteoblastic proliferation and differentiation to consequently stimulate bone formation in vivo, similar to strontium ions [49, 50]. S-PRG filler could continue long-term multiple ion release [12], therefore, tooth surfaces coated with S-PRG nanofiller may acquire long-term antibacterial, anti-inflammatory, and bone-forming effects to reduce periodontitis and restore periodontal health.

## 4 Conclusion

We assessed the antibacterial effects of S-PRG nanofiller coating in vitro. SEM and EDX images showed stable tooth coating with S-PRG nanofillers. Dentin blocks coated with S-PRG nanofiller exhibited bactericidal and growth inhibitory effects against oral bacterial cells related to biofilm formation. In addition, we examined the effects of S-PRG nanofiller coating on clinical parameters of periodontitis in dogs. S-PRG nanofiller coating improved inflammatory parameters, such as GI and BOP, reduce PPD and CAL, and enhance bone healing. Therefore, antibacterial S-PRG nanofiller coating of the tooth surface would be beneficial for periodontal treatment.

## Declarations

### Author contribution statement

Kayoko Mayumi, Hirofumi Miyaji: Conceived and designed the experiments; Performed the experiments; Analyzed and interpreted the data; Contributed reagents, materials, analysis tools or data; Wrote the paper.

Saori Miyata: Analyzed and interpreted the data; Contributed reagents, materials, analysis tools or data.

Erika Nishida, Tomokazu Furihata, Yukimi Kanemoto, Kanako Shitomi: Performed the experiments; Contributed reagents, materials, analysis tools or data.

Tsutomu Sugaya: Analyzed and interpreted the data.

Tsukasa Akasaka: Performed the experiments; Analyzed and interpreted the data.

### Funding statement

This work was supported by the translational research program of the 10.13039/100009619Japan Agency for Medical Research and Development (AMED, 2015-A40).

### Data availability statement

Data included in article/supplementary material/referenced in article.

### Competing interest statement

The authors declare no conflict of interest.

### Additional information

No additional information is available for this paper.
