# Supplementary material for: Antibacterial coating of tooth surface with ion-releasing pre-reacted glass-ionomer (S-PRG) nanofillers
Source: Heliyon. 2021 Feb 2;7(2):e06147. doi: 10.1016/j.heliyon.2021.e06147 (PMC7889979; doi:10.1016/j.heliyon.2021.e06147)
Supplement: 210123 Supplementary figures [file mmc1.pdf]

Supplementary information

Antibacterial coating of tooth surface with ion-releasing pre-reacted glass-ionomer (S-PRG) nanofillers

Kayoko MAYUMI[1], Hirofumi MIYAJI[1]*, Saori MIYATA[1], Erika NISHIDA[1], Tomokazu FURIHATA[1], Yukimi KANEMOTO[1], Tsutomu SUGAYA[1], Kanako SHITOMI[2], Tsukasa AKASAKA[3]

[1] Department of Periodontology and Endodontology, Faculty of Dental Medicine, Hokkaido University, N13 W7, Kita-ku, Sapporo, Hokkaido 060-8586, Japan

[2] Division of Periodontology and Endodontology, School of Dentistry, Health Sciences University of Hokkaido, 1757 Kanazawa, Tobetsu-cho, Ishikari-gun, Hokkaido 061-0293, Japan

[3] Department of Biomedical Materials and Engineering, Faculty of Dental Medicine, Hokkaido University, N13 W7, Kita-ku, Sapporo, Hokkaido 060-8586, Japan

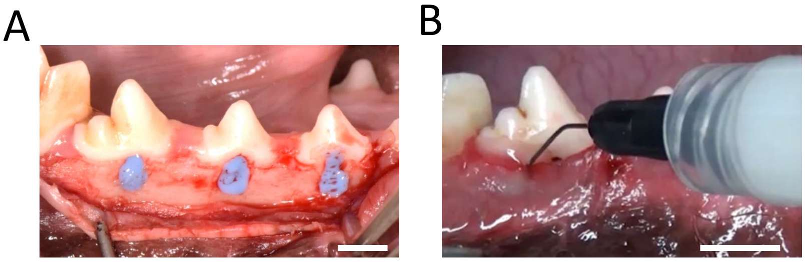

Fig. S1. Surgical procedure and S-PRG nanofiller coating

(A) Digital photographs of experimental furcation defect filled with impression material. (B) Application of S-PRG nanofiller to tooth root surface using a syringe. Scale bar = 1 mm. S-PRG, surface pre-reacted glass-ionomer.

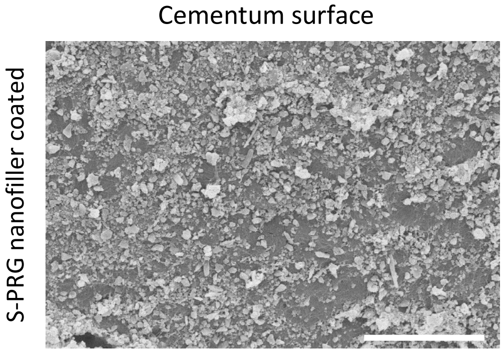

Fig. S2. SEM images of root cementum surface coated with S-PRG nanofillers. Scale bar = 10 µm. SEM, scanning electron microscopy; S-PRG, surface pre-reacted glass-ionomer.
